# Immigrant Patients Adapt to the Culture of Admission and Experience Less Safety in Forensic Psychiatric Care

**DOI:** 10.3389/fpsyg.2021.701544

**Published:** 2021-07-26

**Authors:** Larissa Titze, Julia Gros, Michael Büsselmann, Maximilian Lutz, Judith Streb, Manuela Dudeck

**Affiliations:** Department of Forensic Psychiatry and Psychotherapy, University of Ulm, Ulm, Germany

**Keywords:** acculturation, migration background, forensic psychiatry, ward climate, integration, experienced safety

## Abstract

Patients with an immigrant background are overrepresented in forensic psychiatric hospitals. As a result, daily work is impeded by language barriers and cultural differences. Furthermore, general therapy processes have not yet been adapted to this special patient population, and little reliable knowledge is available. All immigrants go through an acculturation process, which is related to their mental well-being. Four acculturation strategies exist: integration, separation, assimilation, and marginalization. The strategy chosen depends on the extent of someone’s orientation toward their country of origin and the country of admission. The current study aimed to expand knowledge of forensic patients with a migration background in Germany by evaluating their self-reported acculturation processes and associated individual and social factors, e.g., the ward climate. Therefore, we studied forensic patients with a migration background from 11 forensic hospitals in Bavaria, Germany. Besides completing the Frankfurter Acculturation Scale (FRACC) and Essen Climate Evaluation Schema (EssenCES), the participants provided information on their clinical and biographical history. We recruited 235 patients with a migration background and found that the participants oriented themselves more toward the culture of admission and less toward the country of origin than the reference sample did. Moreover, the patients experienced significantly less safety on the ward than the forensic reference sample did. A possible explanation for the patients’ orientation is the lack of possibilities to adhere to their cultural traditions. Patients may feel less safe because of their limited knowledge of German and cultural misunderstandings.

## Introduction

Germany has developed into an immigration country through various cycles of immigration, i.e., the influx of guest workers in the 1960s and 1970s, the ethnic German late repatriates from countries such as Russia between 1988 and 2000, refugees from the former Yugoslavia since 1993, and refugees from Syria and elsewhere since 2014 (Krahl and Steinböck, 2015; Statistisches Bundesamt, 2020). By 2019, the proportion of people with a migration background had increased up to roughly one fourth (25.5%; 20.8 million) of the general German population (Statistisches Bundesamt, 2020).

Surveys show that migrants are overrepresented in forensic psychiatric hospitals compared to the general population. Bulla et al. (2018) report a rate of 35.6% for the state of Baden-Württemberg, while the proportion of migrants in the general population in this state was 28% in the same year, 2015 (Statistisches Landesamt Baden-Württemberg, n.d.). Already data from the years 1990–1999 showed increased proportions of patients with a migration background in forensic psychiatry compared to the general population (Hoffmann, 2006).

The goal of forensic psychiatric hospitals is to protect the general population by enhancing the mental health and well-being of patients so that they can be reintegrated safely into society (Müller et al., 2017). Thus, patients require appropriate medical attention and therapy. Patient needs can vary significantly depending on their diagnosis and the reason for their treatment. In general, patients in German forensic institutions are accommodated under one of two sections of the German criminal code, Section 63 and 64 (Müller et al., 2017). Section 64 is the more frequently used one and applies to patients whose criminal offense was influenced by drug abuse or -dependence and is limited to two years. Section 63 applies to patients who were found to have no or diminished responsibility for their offense because of a lack of insight and/or accountability due to mental illness, profound disturbance of consciousness, intellectual deficiency, or another mental issue (Section 20, 21 of the German criminal code). Whereas patients sentenced under Section 64 of the German criminal code all have a diagnosis of substance abuse or dependence, often combined with a comorbid disorder, patients sentenced under Section 63 have a much larger range of diagnoses, such as schizophrenia spectrum disorder, personality disorders, and learning deficits. Consequently, the therapeutic programs differ between and within wards for patients sentenced under the two sections.

Cultural background affects patient groups to a different extent, and insight and perspectives on psychological well-being differ not only between cultures but also between religions and psychological disorders. For instance, a study by Angermeyer et al. (2016) showed differences between North African and German perspectives and stigmas toward schizophrenia spectrum disorder. In national population surveys, Tunisians ascribed the disorder to psychosocial causes, whereas Germans believed that the cause was found in the brain. Moreover, Tunisians believed more frequently that the affected person bears responsibility for the onset of and recovery from the illness and that a normal life is possible after recovery. Furthermore, the inhabitants of the two countries differed in the amount of contact they desired with people with psychosis. Interestingly, Tunisians made a clear distinction between distant relationships (e.g., if the affected person is a neighbor or co-worker) and family-related relationships. In contrast, Germans reported wanting the same amount of social distance independent of the type of relationship. Overall, Angermeyer et al. (2016) showed that the two cultures differ not only in their perspective on the illness itself, including onset and recovery, but also in their stigmas and behavior toward affected persons. Hence, cultural differences seem important for psychoeducation of patients, for preparing them for reintegration into their family and society and for their associated expectations.

As mentioned above, Germany has been a destination for immigrants for several decades. People immigrate for a variety of reasons. In general, the empirical literature defines three forms of migration (Liedl et al., 2017): (1) migration can be perceived as an opportunity for better employment or life circumstances, e.g., the guest workers who migrated to Germany from Italy in the 1960s and 1970s; (2) migration can happen as a self-decided reaction to crisis situations, e.g., the refugee movements from Syria; and (3) migration can be involuntary, violent, and forced, e.g., the expulsion of the Rohingya from Myanmar. Thus, the reason for and experience of migration can vary greatly between immigrants, as can the associated challenges and stresses they experience. For instance, immigrants who are forced to leave their country because of life-threatening circumstances are more likely to have experienced traumatic events and thus are more vulnerable for developing mental health problems such as posttraumatic stress disorder (Steel et al., 2009). Overall, immigrants often have difficulties achieving socioeconomic stability and establishing social networks, which can also affect their mental health (Hofmeister, 2014).

**FIGURE 1:**
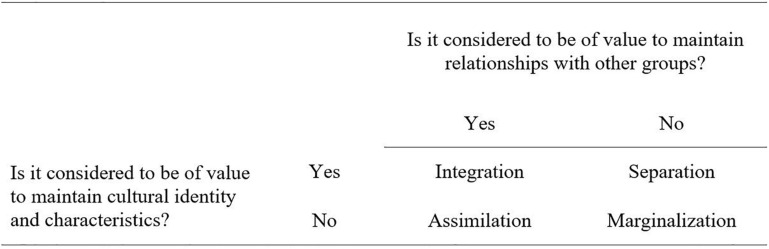
Model of four acculturation strategies. Adapted from Berry (1992).

After leaving the country of origin, the migration process continues with the acculturation process in the culture of admission. The prevailing acculturation model published by Berry (1990) proposes four different strategies with which an immigrant may acculturate to a new culture (see Figure 1): (1) *assimilation* describes the process in which an immigrant completely adapts to the host culture and gives up the values of the culture of origin; (2) *integration* describes the combination of adjusting to the new culture while adhering to the norms of one’s culture of origin; (3) *separation* is the demarcation from the host culture and maintenance of the principles of the culture of origin; and (4) *marginalization* is the complete isolation from both the host culture and the culture of origin. Acculturation processes can lead to extremely high stress levels, also called “acculturation stress” and thus are related to immigrants’ psychological well-being (Berry, 1994; Schmitz, 1994; Haasen et al., 2007).

Overall, for immigrants living in the general population, integration is associated with better psychological well-being than the other three strategies (Berry, 2006; Sam and Berry, 2006; Berry and Sabatier, 2010; Ince et al., 2014; Han et al., 2016). Selten and Cantor-Graae (2005) explain that integrated individuals have more cultural resources to rely on when coping with stress and anxiety.

By the time immigrants arrived in the country of admission, nothing can be done about the act of or reason for migration. However, the acculturation process is still adaptable, so more research is needed to identify contributing factors that may facilitate the adaptation phase and increase mental resilience. Especially in forensic psychiatric hospitals, where mental health is the top priority, knowing the role acculturation processes play is of great importance. The empirical literature on psychotherapy with patients with a migration background clearly recommends that therapists are culture- aware and knowledgeable about the different cultural perspectives on mental health and therapy (Machleidt, 2002; Behrens and Calliess, 2008; Liedl et al., 2017). Hence, newly gained information on acculturation strategies and related factors in forensic patients can be used to design therapy and rehabilitation plans that take acculturation processes into account.

Having a variety of cultural backgrounds, languages, and therapy perspectives on a forensic psychiatric ward takes a toll on the ward atmosphere (Hoffmann, 2006). In a study of prison inmates, Lutz et al. (2019) showed significantly more distress in migrants who had poor or no social relationships with fellow inmates and in those who were more afraid of experiencing crime. Interestingly, migrants in that study stated that they were less likely than native citizens to be victims of crimes in prison, such as being blackmailed, robbed, humiliated, sexually abused, or raped. Thus, even though migrants experienced less crime, they had a greater fear of it. According to the authors, one possible explanation might be that migrants take violence personally and interpret it as a kind of discrimination whereas native citizens attribute conflicts with others to the rough living conditions in prison.

Because few studies have examined the process and effects of acculturation in patients with a migration background in forensic psychiatric hospitals, the current study aimed to explore both the acculturation strategies of such patients and immigrants’ perception of the social atmosphere on the ward in forensic institutions in Bavaria, Germany.

## Materials and Methods

### Participants

Participants were forensic inpatients with a migration background. They were recruited between May 2020 and February 2021 from eleven different forensic psychiatric hospitals in the state of Bavaria, Germany.

### Materials

#### Assessment of Sociodemographic, Clinical, and Legal Data

Participants provided sociodemographic, clinical, and legal information by completing a self-report questionnaire. The questionnaire comprised questions about age, sex, education, nationality, parents’ nationality, residence status, current residence, the criminal code under which their placement in forensic psychiatry had been ordered, diagnoses, index offense and criminal record.

#### Frankfurt Acculturation Scale

This German scale by Bongard et al. (2020) captures acculturation strategy, according to the model from Berry (1990), on the two scales “orientation to the culture of origin” (OC) and “orientation to the culture of admission” (AC). For the purpose of constructing, analyzing, retesting and norming, 22 different subsamples have been aggregated to 4 main samples. The construction sample consisted of 305 first-generation migrants (52% female; *M*_age_ = 38.3 years, SD_age_ = 14.5 years), from Bosnia, Iran and South Korea. The sample for analyses contained 2,516 participants from over 45 different countries (59% female; *M*_age_ = 38.3 years, SD_age_ = 13.1 years). The retest-sample included 175 first- and second-generation migrants with different nationalities (61% female; *M*_age_ = 33.4 years, *SD*_age_ = 12.6 years). The retest-reliability was tested at intervals of two, four, and six weeks. The last main sample was the reference-sample. Therefore 3,079 first- and second-generation immigrants to Germany (61% female; *M*_age_ = 37.1 years, *SD*_age_ = 12.9), mostly from Turkey, Poland, Bulgaria, Iran, Bosnia, Morocco, Korea, and Latin America were evaluated.

The questionnaire consists of 20 questions, that are answered on a seven-point Likert scale (ranging from 0 = completely incorrect to 6 = completely correct). To evaluate the responses, the sum of each scale is calculated, and the dominant acculturation strategy (integration, assimilation, separation, or marginalization) is determined by a median-split technique. To interpret the results, we compared them with the results of the norming-sample reported by Bongard et al. (2020). Bongard et al. (2020) found good internal consistency (HC, α = 0.86 and AC, α = 0.85) and retest-reliability (HC, *r_*tt*_* = 0.86, and AC, *r_*tt*_* = 0.73) of the scale.

#### Essen Climate Evaluation Schema

This questionnaire, which was developed for forensic psychiatry contains 15 items that measure the ward climate with three scales: therapeutic hold (α = 0.86), patients’ cohesion and mutual support (α = 0.78), and experienced safety (α = 0.79; Schalast and Tonkin, 2016). The scale therapeutic hold contains items like “staff know patients and their personal histories very well” (Schalast and Tonkin, 2016) and measures how stable the therapeutic relationship is and if the staff is devoted to the patients (Schalast, 2008). “The patients take care of each other” (Schalast and Tonkin, 2016) is an example for an item of the patients’ cohesion and mutual support-scale, which measures the existence of a therapeutic companionship (Schalast, 2008). And the third subscale experienced safety can detect how personally safe the patients feel, contrary to go through aggressiveness or violence. An example is: “there are some really quite threatening situations here.” The items are rated on a five-point Likert-scale ranging from 0 (completely incorrect) to 5 (completely correct). For the analysis, sum scores are computed and interpreted by means of quintiles, which have been normed on the basis of 1,302 patients of 102 wards across 31 German forensic psychiatric hospitals. The wards had different security levels and the patients were detained according to section 63 or 64 of the German Penal Code. The factor structure was verified for forensic samples, and the convergent validity was supported by moderate correlations (*r* = 0.47–0.78; Schalast and Tonkin, 2016).

### Procedure

The study was approved by the Ethics Committee of the University Ulm, Germany (approval no. 145/19). We then contacted all forensic psychiatric institutions in Bavaria, Germany, informed them about the content and aims of the study and asked whether they were interested in participating. If they agreed to participate, the contact person was asked to provide anonymized advance information about the patients (e.g., native language, country of origin, whether patients are literate, etc.) via e-mail. This information was used to prepare the study materials. Translations of the questionnaires were made by a professional translating agency, Toptranslation GmbH, Hamburg, Germany. Afterward, appointments for data collection were agreed upon with the institutions.

For data collection, research assistants visited the wards at the institutions, which provided a separate room for testing. Patients were informed orally about the research project and told about the applicable privacy policies and that participation was voluntary. They were given a written information sheet and sufficient time to ask questions and decide whether they were willing to participate. If patients were not literate in German but were literate in their native language or another language, they were given the information sheet, informed consent form, and questionnaires written in that language. 20% (*n* = 47) used the material in a translated version, mostly English, Persian and Arabic, therefore 80% (*n* = 188) were able to conduct the study in German. For patients who were not literate in German, their native language, or any other language, all documents were read out loud by the research assistants in German or English. This method was used in only 14% (*n* = 33) of the cases, 86% (*n* = 202) completed the questionnaires themselves in written form. Patients who were not literate in any language and did not speak or understand any spoken German or English were excluded from participation. Participants received the questionnaires only after signing the informed consent form and were able to withdraw their consent to participate at any time without giving a reason.

### Data Analysis

Data were analyzed with IBM SPSS Statistics for Windows Version 27 (Armok, NY, United States, IBM Corp.). For descriptive statistics, absolute and relative frequencies, mean values, standard deviations, and ranges were calculated. Crosstabs were used to generate contingency tables and describe interactions. The current sample was compared with the sample provided in the manuals by a one-sample *t*-test, and subgroups were compared by *t*-tests for independent samples. Differences in duration of hospital stay, years in Germany, age and quotient of years in Germany/age between patients with different acculturation strategies (marginalization, separation, integration, and assimilation) were analyzed using the Kruskal–Wallis test. Further, Spearman-correlations were computed between the orientation toward the country of admission/origin and the duration of hospital stay, years in Germany, age and the quotient of years in Germany by age.

## Results

The sample consisted of 235 forensic inpatients with a migration background, including 186 first generation immigrants, who were not born in Germany and 49 second generation immigrants, whose parents or at least one parent was not born in Germany. A total of 60 men and 3 women were detained according to Section 63 of the German penal code (patients with severe mental disorders), and 150 men and 7 women were detained according to Section 64 (patients with substance use disorders). The sociodemographic and forensic-psychiatric characteristics of the participating patients with migration background can be seen in Table 1.

**TABLE 1:** Sociodemographic and forensic-psychiatric characteristics of the participants with a migration background.

	**Statistics**
Age^1^, *M* (*SD*; range), (years)	33.1 (8.37; 19–64)
Years at school^2^	9.4 (3.42; 0–18)
**Years in Germany^1^, *M* (*SD*; range)**	
*First-generation immigrants*	13.8 (10.55; 1–53)
*Second-generation immigrants*	29.8 (7.38; 13–48)
Years in Germany/age^3^ *M* (*SD*; range) *First-generation immigrants Second-generation immigrants*	0.40 (0.28; 0.03–1.00) 0.98 (0.07; 0.59–1.00)
**Native language, *n* (%)**	
*Russian*	41 (17)
*Turkish*	26 (11)
*Arabic*	27 (12)
*Persian*	18 (8)
*Romanian*	15 (6)
*German*	15 (6)
*Albania*	13 (6)
*Polish*	13 (6)
*Other language (35 languages, each < 4%)*	67 (29)
Further language skills, *M* (*SD*; range)	2.0 (1.19; 0–7)
Duration of hospital stay^4^ (months)	15.3 (25.25; 0.5–240)
**Diagnosis, n (%)**	
*Substance use disorder*	147 (62)
*Schizophrenic disorder*	46 (19)
*Personality disorder*	20 (8)
*Other diagnosis*	24 (10)
**Index offense^5^, *n* (%)**	
*Homicide*	13 (6)
*Robbery*	25 (11)
*Aggravated assault*	73 (32)
*Sexual assault*	13 (6)
*Fraud/Theft*	14 (6)
*Arson*	5 (2)
*Violation of the Narcotics Act*	74 (33)
*Other offenses*	9 (4)

*^1^Missing data: *n* = 1. ^2^Missing data: *n* = 5. ^3^Missing data: *n* = 2. ^4^Missing data: *n* = 13. ^5^Missing data: *n* = 11.*

### Acculturation

**FIGURE 2:**
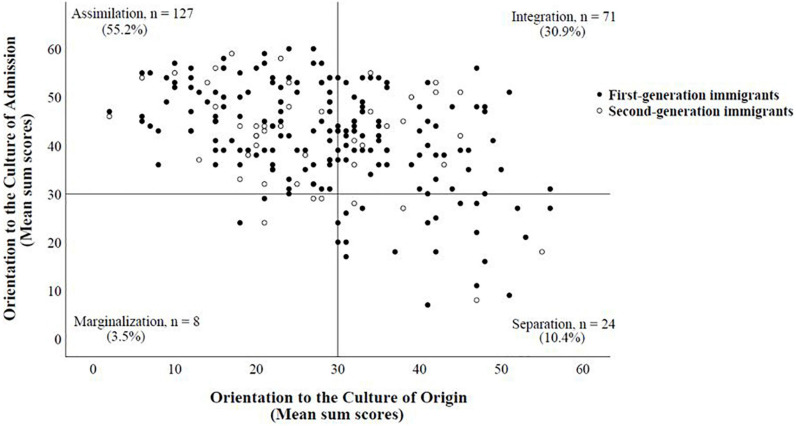
Distribution of acculturation strategies among forensic psychiatric patients with a migration background (*N* = 230^1^). ^1^Missing values: *n* = 5. Every dot represents the mean sum score of one patient, and the reference lines represent the center of the subscales on the Frankfurter Acculturation Scale (FRACC). The distance between a dot and the reference line reflects the strength of the respective acculturation strategy, i.e., the greater the distance, the stronger the respective acculturation strategy.

No differences were found between patients detained according to Section 63 or 64 concerning their acculturation strategy (Fishers exact test, *p* = 0.541), their orientation toward Germany (*t* (215) = −1.49, *p* = 0.138, *M_*t_*__63_* = 50.1 vs. *M_*t_*__64_* = 52.3), or their orientation toward their country of origin (*t* (216) = 1.19, *p* = 0.236, *M_*t_*__63_* = 43.7 vs. *M_*t_*__64_* = *42.0*). Compared with persons in the above-mentioned reference sample of Bongard et al. (2020), the patients of the current sample oriented themselves more toward the country of admission (*t* (229) = 2.46, *p* = 0.015, *M*_*t*_ = 51.6 vs. *M*_*t,Bongard*_ = 50.0) and less toward the country of origin (*t* (230) = −12.04, *p* < 0.001, *M*_*t*_ = 42.4 vs. *M*_*t,Bongard*_ = 50.0). Acculturation strategies were not different between first- and second-generation immigrants [Chi^2^ (3) = 1.18, *p* = 0.759]. The acculturation strategies of the sample are shown in Figure 2.

As can be seen in Table 2 patients with different acculturation strategies did not differ with regard to the duration of hospital stay, years in Germany, age and with regard to the quotient years in Germany/age. There were also no significant correlations between their orientation toward Germany and the duration of hospital stay (*r_*s*_* = −0.02), years in Germany (*r_*s*_* = 0.12), age (*r_*s*_* = 0.03), or the quotient years in Germany by age (*r_*s*_* = 0.10), nor between their orientation toward their country of origin and the duration of hospital stay (*r_*s*_* = 0.03), years in Germany (*r _*s*_* = −0.08), age (*r_*s*_* = 0.05), or the quotient years in Germany by age (*r_*s*_* = −0.06).

**TABLE 2:** Differences in duration of hospital stay, years in Germany, age and quotient of years in Germany/age between patients with different acculturation strategies (marginalization, separation, integration, and assimilation).

	**Marginalization *M* (*SD*)**	**Separation *M* (*SD*)**	**Integration *M* (*SD*)**	**Assimiliation *M* (*SD*)**	**Statistics Kruskal–Wallis-*H df = 2***
Duration of hospital stay (months)	16.89 (15.36)	15.08 (10.42)	15.89 (10.77)	18.09 (12.52)	2.32 *p* = 0.509
Years in Germany	11.50 (9.34)	16.15 (25.01)	19.70 (37.20)	13.11 (16.60)	0.31 *p* = 0.959
Age (years)	34.00 (8.32)	32.54 (7.03)	32.34 (9.06)	33.48 (8.19)	1.10 *p* = 0.573
Quotient (years in Germany/age)	0.52 (0.43)	0.48 (0.36)	0.50 (0.33)	0.54 (0.36)	1.05 *p* = 0.790

### Ward Climate

**FIGURE 3:**
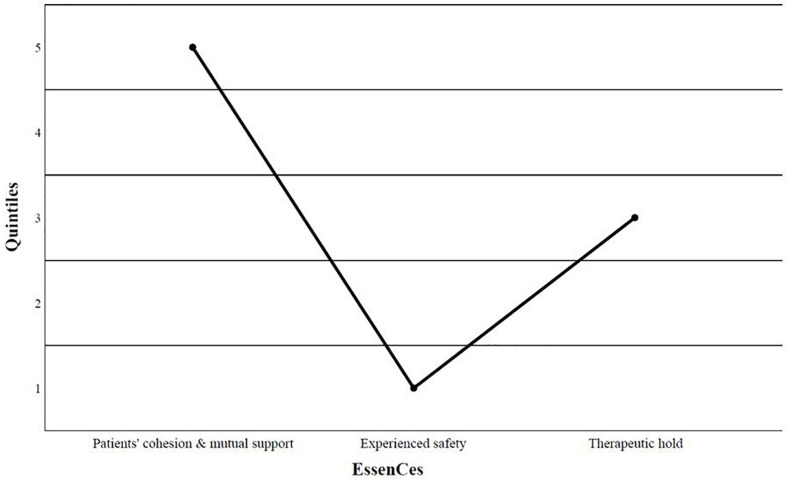
Classification of the ward climate according to the qiiintiles of the forensic reference sample of Schalast and Tonkin (2016), (*N* = 236). Q1 = clearly below average, Q2 = somewhat below average, Q3 = average, Q4 = somewhat above average, Q5 = clearly above average.

The *t*-tests revealed no significant differences between patients detained according to Section 63 or 64 in the three subscales of the Essen Climate Evaluation Schema (EssenCES): therapeutic hold [*t* (216) = 0.83, *p* = 0.41, *M*_63_ = 12.4 vs. *M*_64_ = 12.0], patients’ cohesion and mutual support [*t* (216) = 1.71, *p* = 0.088, *M*_63_ = 12.2 vs. *M*_64_ = 11.1], and experienced safety [*t* (216) = 0.32, *p* = 0.752, *M*_63_ = 6.3 vs. *M*_64_ = 6.1]. We compared the mean ratings of our sample with the quintiles of the forensic reference sample mentioned above (Schalast and Tonkin, 2016). In the current sample, therapeutic hold was rated as average, and patients’ cohesion and mutual support were rated as clearly above average; in contrast, experienced safety was rated as clearly below average (Figure 3).

Statistical analyses confirmed the initial impression: Patients in our sample experienced safety to a significantly lower degree than patients in the reference sample [*t* (230) = −22.2, *p* < 0.001, *M* = 6.2 vs. *M*_Norm_ = 13.1]. The rating of patients’ cohesion and mutual support was significantly higher in our sample than in the reference sample [*t* (230) = 3.61, *p* < 0.001, *M* = 11.4 vs. *M*_Norm_ = 10.4], but that of therapeutic hold was not different between the two samples [*t* (230) = 0.42, *p* = 0.673, *M* = 12.1 vs. *M*_Norm_ = 12.0]. First- and second-generation immigrants evaluated the scale therapeutic hold differently in that the former rated it more positively than the latter [*t* (230) = 2.88, *p* = 0.004, M_1__st_ = 12.4 M_2__nd_ = 10.9]. No differences were found in the other two scales [patients’ cohesion and mutual support: *t* (230) = 0.29, *p* = 0.769, M_1__st_ = 11.5 vs. M_2__nd_ = 11.3; experienced safety: *t* (230) = 0.23, *p* = 0.817, M_1__st_ = 6.3 vs. M_2__nd_ = 6.1].

## Discussion

In this study, we explored both the acculturation strategies of patients with a migration background and patients’ perception of the social atmosphere on the ward in forensic psychiatric hospitals in Bavaria, Germany. Our findings are discussed below.

### Acculturation

Patients in our study were oriented more toward the country of admission and less toward the country of origin as compared with the reference sample. A possible explanation for this difference may be the different living circumstances of inpatients with a migration background and persons with a migration background in the general population. For instance, patients in forensic institutions have limited freedom to follow their cultural traditions and customs. E.g., the preparation of traditional meals is complicated by the fact that patients often receive prepared meals or do not have access to the full range of foods. Furthermore, research showed that mass media may not only facilitate adaptation processes by importing norms and values of the host culture but also help maintain a cultural connection with the culture of origin (Barnett and McPhail, 1980). However, patients in forensic institutions, can mostly only watch German TV programs or read German newspapers; hence, they are predominantly exposed to German culture and have fewer opportunities to connect with their heritage culture. Moreover, the frequency of cross-cultural contact was shown to be predictive of the adaptation to the host culture (Clément, 1986). Although immigrants in the general population may be able to live undisturbed in subcultures, patients in institutions such as forensic psychiatric hospitals are forced to engage in the new culture and are at least approached in the language of the country of admission. Consequently, the inevitable contact with the host culture and the decreased opportunity to adhere to cultural traditions may result in inpatients with a migration background adapting more to the host culture than their counterparts living in the general population.

### Ward Climate

The level of safety experienced by the patients in our sample was clearly below average compared with the forensic reference sample. In general, people tend to feel less safe, when they are unable to speak the language of the host country properly (Kearns and Whitley, 2015). Thus, patients with a migration background may not be able to understand, or explain a situation appropriately, and if necessary, defend themselves adequately. However, this theory does not fully explain our results, because we found no differences in the assessment of safety between first- and second-generation immigrants. Therefore, not only language difficulties but also culture-related misunderstandings could contribute to the patients feeling less safe (Kalengayi et al., 2016). These misunderstandings are the result of the underlying difficulties of living in a foreign cultural environment. Moreover, changes of culture *per se* increase migrants’ overall stress (Bustamante et al., 2018) and could therefore lead to insecurity. Furthermore, racist experiences cannot be ruled out (Bhatia, 2020); institutional racism acts as a stressor (Bhugra, 2000) and may result in less experienced safety. In order to find out whether the lower experienced safety is due to cultural misunderstandings, qualitative interviews should be conducted with the patients. In doing so, the interviewers could also elicit whether and, if so, how often patients are exposed to racist experiences. In the literature it is described that the security level of psychiatric hospitals is another factor influencing individual experiences of safety (Efkemann et al., 2019). Efkemann et al. (2019) examined hospitals, with different ward settings and door policies, and were able to show that involuntarily committed patients rated the EssenCES’ subscale “experiences safety” higher in an open setting compared to a facultatively locked and a locked setting. Others examined the impact of patient characteristics and were able to show that female gender or diagnosis of personality disorders or psychosis are associated with higher scores of experienced safety (Dickens et al., 2014). These factors should be considered in further studies with migrants.

Patients’ cohesion and mutual support reported by patients with a migration background was significantly higher than in the reference sample and was clearly above average. This finding may be explained by a lack of trust of migrants toward official services and foreign health services as a result of their experiences (Priebe et al., 2016), which means that they may have to rely more on fellow patients. To test these hypotheses, patients’ reasons for high cohesion would need to be explicitly asked. Another reason for the high scores in the patients’ cohesion scale could also be that patients with a migration background seek more contact with fellow patients and form more friendships than others because their society of origin is oriented more toward collectivism in contrast to individualistic societies like Germany. This question could be answered in the context of a survey exploring the degree to which people in a society are integrated into groups (Hofstede, 1993). There was no difference in the measures of the therapeutic hold, meaning that patients with a migration background feel as supported by the therapists as the norm sample. This could be taken as a sign that therapists want to give every patient the same level of support, regardless of their migration background.

### Limitations

This study has some limitations. First, all data were collected via self-report, which is likely to be influenced by social desirability bias and amnestic disorders. Social desirability is an important issue in studies conducted in forensic psychiatry and even more so in studies with persons with a migration background (Heeren et al., 2016). Even if participants were informed numerous times that the study was pseudonymized and that no personal data will be published, patients frequently expressed the fear that their answers will become known to the treatment team or other officials. Furthermore, the questionnaires used in the study posed a challenge for some of the patients, e.g., the Frankfurter Acculturation Scale (FRACC) seven-point Likert-scale was difficult to understand and participants therefore sometimes simplified it by only using 0 (completely incorrect), 3 (neither here nor there) and 6 (completely correct).

### Recommendations for Future Studies

Further research is necessary to clarify the mechanisms behind the findings, as the interpretations mentioned, can only be considered as suggestions. For future studies it would be useful to simplify the questionnaires, as the seven-point Likert-scale was sometimes too difficult. Furthermore, the support of translators would be beneficial. On the one hand, to standardize the procedure and on the other hand, also patients with no alphabetization or oral German or English knowledge, could be interviewed then. To clarify the questions if either language or cultural misunderstandings are the reason for the unsafe feeling of patients with a migration background and if these patients rely more on other patients because of a lack of trust in officials, tailored questions should be added in future studies.

## Conclusion

Studies on acculturation stress research showed that a balanced relationship between orientation to the culture of origin and the host culture is to be strived for (i.e., *integration* in the above-mentioned model from Berry, 1990). Therefore, therapists and caregivers should help migrants to integrate rather than just assimilate because maintaining ties to their culture of origin can also give patients security and support. It is important not only to accompany patients on their journey from the culture of origin to the host culture but also to help ensure that resources from both cultures are combined. Thus, patients with a migration background should form a bicultural identity. Concrete approaches to the integration of migrants in psychiatric care have been formulated by various authors (Machleidt, 2002; De Jong and Van Ommeren, 2005); however, their feasibility in forensic psychiatry would have to be examined in a next step.

Evaluating the current conditions on a forensic ward with a social climate scale does not automatically bring about any improvement by itself. However, it may be a starting point for discussing the situation and may help to mitigate problems of migrants in forensic psychiatry. Training that prepares staff to de-escalate critical situations has been shown to be valuable because it can encourage greater feelings of safety amongst patients (Hollin and Howells, 1989). Another beneficial measure may be for staff to systematically explore ways of helping patients with a migration background settle conflicts (Schalast and Tonkin, 2016).

## Data Availability Statement

The raw data supporting the conclusions of this article will be made available by the authors, without undue reservation. Requests to access the datasets should be directed to JS, judith.streb@uni-ulm.de.

## Ethics Statement

The studies involving human participants were reviewed and approved by Ethics Committee of the University Ulm, Germany. The patients/participants provided their written informed consent to participate in this study.

## Author Contributions

MD and JS designed the study. LT and JG collected the data. LT, JG, and ML analyzed the data. LT, JG, and MB interpreted the data. LT wrote the initial draft of the manuscript. All authors had full access to all the data in the study and took responsibility for the integrity and accuracy of the data analysis, and read and approved the final version of the manuscript.

## Conflict of Interest

The authors declare that the research was conducted in the absence of any commercial or financial relationships that could be construed as a potential conflict of interest.

## Publisher’s Note

All claims expressed in this article are solely those of the authors and do not necessarily represent those of their affiliated organizations, or those of the publisher, the editors and the reviewers. Any product that may be evaluated in this article, or claim that may be made by its manufacturer, is not guaranteed or endorsed by the publisher.
